# Macrophage depletion reduces postsurgical tumor recurrence and metastatic growth in a spontaneous murine model of melanoma

**DOI:** 10.18632/oncotarget.3127

**Published:** 2015-02-13

**Authors:** Muly Tham, Karen Khoo, Kim Pin Yeo, Masashi Kato, Amelle Prevost-Blondel, Veronique Angeli, Jean-Pierre Abastado

**Affiliations:** ^1^ Singapore Immunology Network, BMSI, A-STAR, Singapore; ^2^ Department of Microbiology, Yong Loo Lin School of Medicine, National University of Singapore, Singapore; ^3^ Department of Occupational and Environmental Health, Nagoya University Graduate School of Medicine, Japan; ^4^ Institut Cochin, Université Paris Descartes, CNRS UMR 8104, Paris, France; ^5^ Institut de Recherche Internationales Servier, Suresnes, France

**Keywords:** CSF1R inhibition, Macrophages, Postsurgical relapse, Tumor-initiating cell

## Abstract

Surgical resection of tumors is often followed by regrowth at the primary site and metastases may emerge rapidly following removal of the primary tumor. Macrophages are important drivers of tumor growth, and here we investigated their involvement in postoperative relapse as well as explore macrophage depletion as an adjuvant to surgical resection. RETAAD mice develop spontaneous metastatic melanoma that begins in the eye. Removal of the eyes as early as 1 week of age did not prevent the development of metastases; rather, surgery led to increased proliferation of tumor cells locally and in distant metastases. Surgery-induced increase in tumor cell proliferation correlated with increased macrophage density within the tumor. Moreover, macrophages stimulate tumor sphere formation from tumor cells of post-surgical but not control mice. Macrophage depletion with a diet containing the CSF-1R specific kinase inhibitor Ki20227 following surgery significantly reduced postoperative tumor recurrence and abrogated enhanced metastatic outgrowth. Our results confirm that tumor cells disseminate early, and show that macrophages contribute both to post-surgical tumor relapse and growth of metastases, likely through stimulating a population of tumor-initiating cells. Thus macrophage depletion warrants exploration as an adjuvant to surgical resection.

## INTRODUCTION

Surgical resection has been a standard treatment for solid malignancies for over a century. However, much controversy still surrounds this form of therapy as both beneficial and adverse effects have been reported. Although surgical resection may halt the progression of the cancer in the short term, recurrences can occur from residual tumor cells, even over a decade after the initial surgery [1]. Several factors have been associated with tumor recurrence after surgery, including growth factors in the wound fluid, such as TGFβ, FGF [2], HB-EGF and PDGF [3], as well as surgery-induced hypoxia [4] and oxidative stress [5]. Surgery may also promote metastasis by shedding tumor cells during surgery, and accelerate the outgrowth of already-disseminated metastases, since primary tumors can produce inhibitory molecules that regulate the growth of distant metastases [6–8].

The immune system plays an important role in the regulation of tumor development and progression. Post-surgical immune suppression is associated with recurrence and metastatic outgrowth; natural killer cells in particular are suppressed, in terms of numbers and activity, by surgical trauma, contributing to a pro-tumoral environment [9, 10]. Conversely neutrophils present in post-operative lavage fluid can enhance growth of transplanted tumor cells [11], but the roles of other immune cell populations have yet to be characterized. Macrophage infiltration of tumors is associated with poor prognosis and increased relapse in a number of cancers [12–14]. Macrophages have also been well-documented to promote tumor cell proliferation [15], and while recent reports highlighted a role for macrophages in tumor responses to various types of therapy including chemotherapy, immunotherapy and radiotherapy [16–18], little is known about whether macrophages contribute directly to recurrence and metastases after surgical resection.

In the current study we used a well-characterized murine model of spontaneous melanoma to understand how macrophages contribute to post-surgical relapse and metastatic outgrowth. RETAAD mice are immune-competent and develop uveal melanoma owing to expression of the human *RET* oncogene in melanocytes. Following development of the primary tumor in the eye from around three weeks of age, a rapid and progressive metastatic cascade occurs [19], recapitulating many aspects of human melanoma. Here we found that removal of the eyes prior to macroscopic tumor development did not alter the course of disease progression, but instead accelerated outgrowth of residual tumor cells, as well as of distant metastases. Surgery induced the emergence of a population of tumor cells that were able to respond to macrophage signals to initiate tumor sphere growth *in vitro*, and this capacity may be responsible for the enhanced tumor growth seen after surgery. Postoperative depletion of macrophages significantly reduced tumor regrowth at the primary site and abrogated the surgery-enhanced growth of metastases.

## RESULTS

### Surgical resection enhances primary tumor regrowth

**Figure 1:**
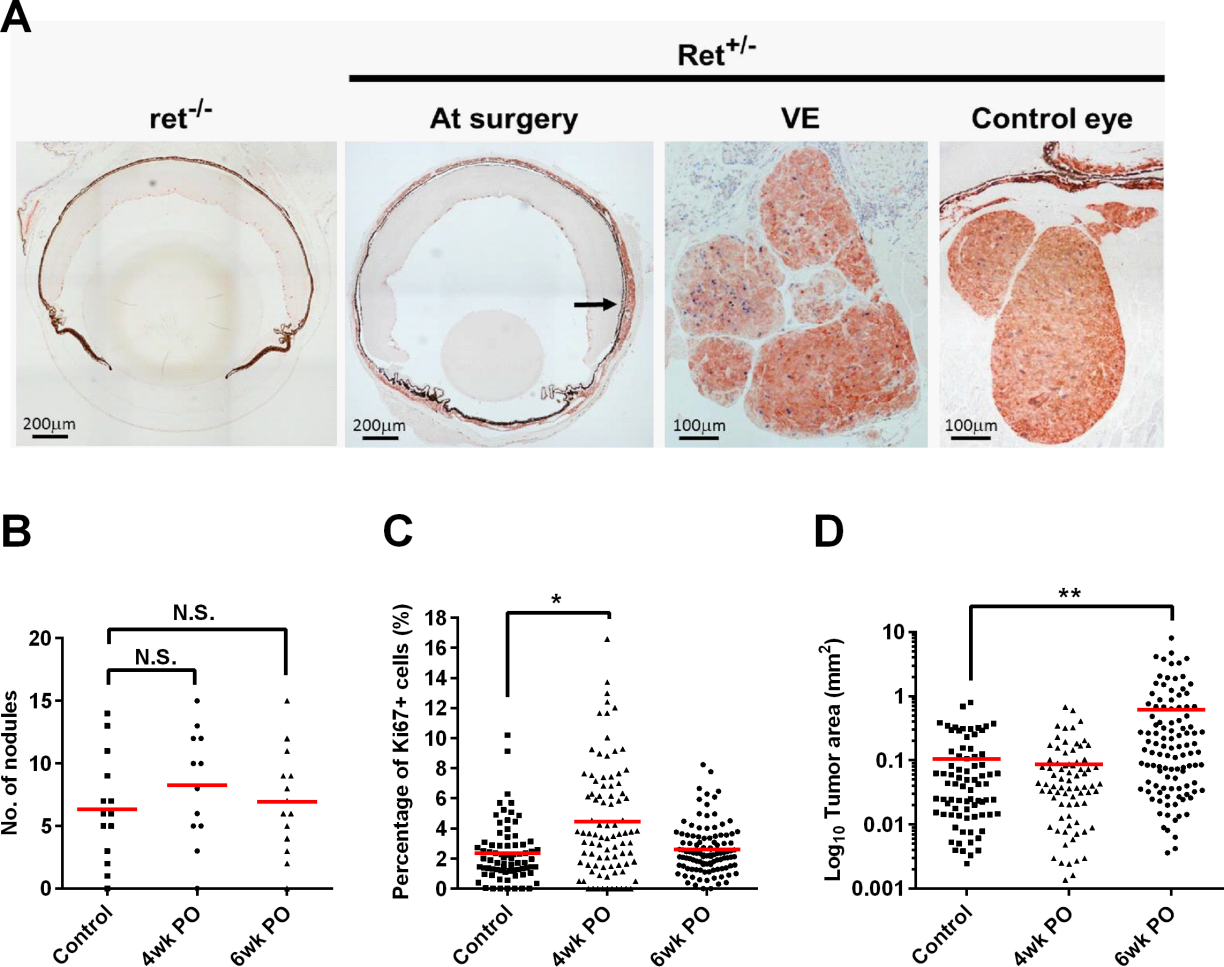
Surgical resection enhances primary tumor regrowth **(A)** IHC labeling of eye sections from non-tumor-bearing (ret^−/−^) and tumor-bearing (Ret^+/−^) mice, with S100B (red) and Ki67 (blue). At the time of surgery (3wk after birth) Ret^+/−^ eyes show signs of hyperplasia (arrow). 4 weeks after surgery (VE), tumor regrowth has occurred and tumors were comparable to those in orbits of control mice. **(B)** Quantification of the number of tumor nodules relapsing within the orbit of the eye 4 and 6wk after surgery. Each point represent one mouse; 1-way ANOVA, N.S. not significant (*n* = 12–14 mice). **(C)** The percentage of Ki67^+^ cells in the relapsed tumors 4 and 6wks after surgery. Each point represents one tumor nodule; 1-way ANOVA, *P < 0.05 (*n* = 4–6 mice). **(D)** Quantification of the area of the relapsed tumor nodules 4 and 6wk after surgery. Each point represents one tumor nodule; 1-way ANOVA, **P < 0.01 (*n* = 3–5 mice).

Many of the studies on surgical resection in cancer have been performed in mice bearing transplanted tumors, which poorly mimic the complexity and natural progression of the disease in humans. Furthermore, transplanted models rarely allow the simultaneous study of a recurring primary tumor and distant metastases in the same animal. In the RETAAD mice used here, the primary tumor can develop in the eye as early as three weeks of age, before metastases become evident in various distant tissues including facial muscles (median onset 66 days), neck/trunk (80 days), reproductive tract (242 days), mediastinum (263 days) and lungs (347 days). The traditional view that “seeding” of distant tissues with metastases occurs late during cancer progression would predict that tumor development and metastasis should be completely abrogated if resection of the eyes (primary tumor development site) was carried out during the early phase of tumor development. We tested this hypothesis: three week old RETAAD pups underwent bilateral visual enucleation (VE) and four weeks later were euthanized and necropsied. Immunohistochemistry on sections of the removed eyes revealed hyperplasia in the choroid layer, even at this young age, seen as increased abundance of cells expressing the melanoma marker S100B compared to ret^−/−^ non-tumor bearing litter mates (Figure 1A), but no tumor nodules were visible. Four weeks after VE, new tumors were present within the eye sockets which were macroscopically comparable to those in non-VE age-matched controls (Figure 1A). Similar results were obtained when we performed surgery in younger animals at one week of age. Thus, early resection of the primary tumor site did not prevent tumor relapse. To evaluate the kinetics of postsurgical tumor regrowth we conducted VE on one group of one week old mice and another group of three weeks old mice, and allowed both to reach seven weeks of age before necropsy and comparison to age-matched non-VE controls. This strategy allowed us to evaluate tumor regrowth at the primary site at both four and six weeks post-operation and minimized the number of control animals required. Following VE a similar number of tumor nodules grew in the eye sockets as in non-VE control mice (Figure 1B). However, tumors from VE mice contained a significantly higher percentage of Ki67-positive proliferating tumor cells compared to controls at four weeks post-operation (4wkPO), but not at 6wkPO (Figure 1C). The average tumor nodule area in VE mice at 6wkPO was significantly greater than in control mice, while tumor area was comparable in both groups at 4wkPO (Figure 1D). This indicates that eye surgical resection increases proliferation of tumor cells locally at 4wkPO, resulting in increased tumor area by 6wkPO.

### Primary tumor resection enhances growth of pre-existing metastases

**Figure 2:**
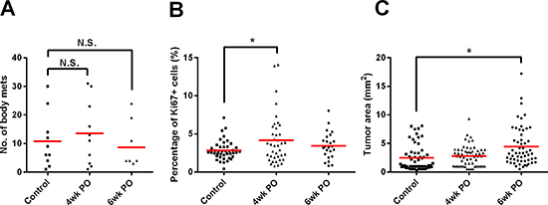
Surgical resection enhances the growth of metastases **(A)** Number of metastases present in mice 4 and 6wk after surgery. Each point represents one mouse; 1-way ANOVA, N.S. not significant (*n* = 8–10 mice). **(B)** The percentage of Ki67^+^ tumor cells present in the metastases 4 and 6wks after surgery. Each point represents one tumor nodule; 1-way ANOVA, *P < 0.05 (*n* = 3–5 mice). **(C)** Quantification of the area of the metastases 4 and 6wk after surgery. Each point represents one tumor nodule; 1-way ANOVA, *P < 0.05 (*n* = 3–7 mice).

The RETAAD mice exhibit a well-defined and reproducible kinetic of cancer development with metastases evident throughout the body soon after initiation in the eyes. Thus we asked whether early removal of the eyes had any effect on the development of metastases. Conducting VE at either one or three weeks of age did not alter the number of metastases detected at necropsy compared to control non-VE mice (Figure 2A), indicating that metastasis was initiated very early, even before detectable outgrowth of the primary tumor. Interestingly, a similar pattern of increased tumor cell proliferation and tumor surface area in metastases of VE mice was seen at 4wkPO (Figure 2B) and 6wkPO (Figure 2C) respectively, as for the tumors at the primary sites in these mice (Figure 1C and 1D). This indicates that surgery results in systemic changes that not only affect the primary tumor/surgical site but also alter metastatic outgrowth.

### Surgery increases macrophage infiltration which correlates with tumor growth

**Figure 3:**
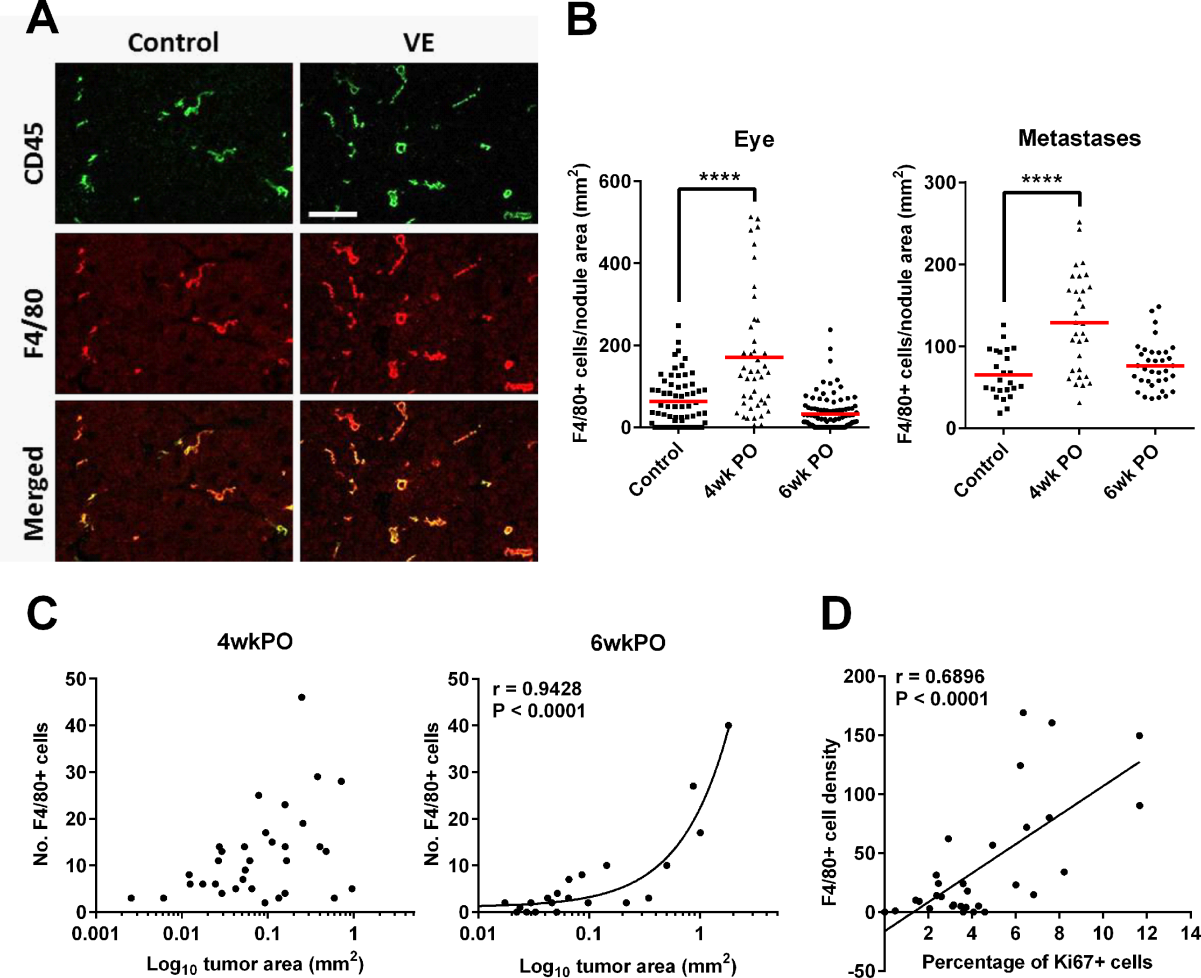
Surgery increases macrophage infiltration which correlates with tumor growth **(A)** Representative immunofluorescence image of tumors from control and VE animals labeled with antibodies against CD45 (green) and F4/80 (red). **(B)** Density of F4/80^+^ TAMs in relapsed eye tumors and metastases at 4 and 6wkPO. Each point represents one tumor nodule; 1-way ANOVA, ****P < 0.0001 (*n* = 3–7). **(C)** Graph showing the correlation (Pearson) of TAM numbers with tumor area at 4wkPO and 6wkPO at the primary site. **(D)** Graph showing the correlation (Pearson) of TAM density and percentage of Ki67^+^ cells within the tumors at 4wkPO at the primary site.

Macrophages normally infiltrate the surgical site to assist wound healing [20]. However, M2/tumor-associated macrophages (TAMs) are also well known to promote tumor growth [21]. We therefore asked whether macrophages contribute to the enhanced local and/or distant tumor growth observed after surgery. We immunolabeled sections of the relapsed primary tumors and of metastases using F4/80 as a marker for TAMs, and found that there was a significantly higher density of TAMs in the tumor bed at 4wkPO, but not at 6wkPO, compared to control non-VE mice (Figure 3A and 3B). These TAMs expressed the M2 marker CD206 on the cell surface, and Arginase 1 and TGFβ at the mRNA level (Supplementary Figure S1A to S1C respectively). The increased number of TAMs at 4wkPO did not correlate with the tumor area at the same time point (Figure 3C left panel), but did at 6wkPO (Figure 3C right panel). Moreover, a close association was seen between TAM density and percentage of Ki67^+^ proliferating cells within the tumors at 4wkPO (Figure 3D). This indicates that M2-type TAMs within the tumors of VE mice are associated with increased tumor cell proliferation at 4wkPO, which results in increased tumor size at 6wkPO.

### Surgery accelerates the emergence of macrophage-responsive tumor-initiating cells

**Figure 4:**
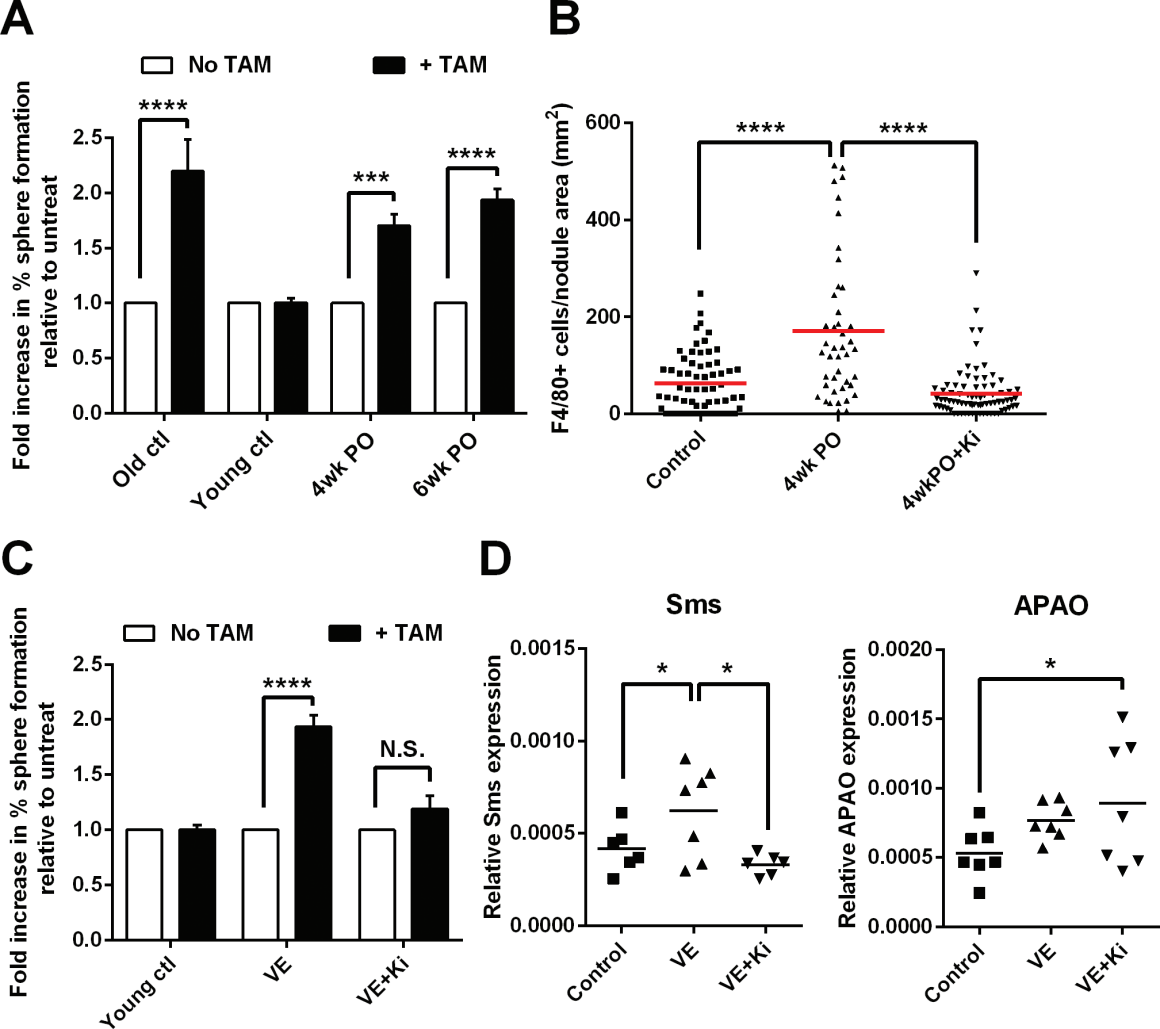
Surgery accelerates the emergence of macrophage-responsive tumor-initiating cells **(A)** Graph showing the fold-change in percentage of cells forming tumor spheres in the presence of TAMs, relative to without TAMs. Tumor cells from young mice (age 10–15 weeks) that underwent VE followed by recovery for 4–6wks were stimulated with TAMs derived from non-VE old mice (> 30wk). Bars represent mean ± SE; 2-way ANOVA, ***P < 0.001, ****P < 0.0001 (*n* = 3–4 mice). **(B)** Density of F4/80^+^ TAMs in primary relapsed tumors from mice that underwent VE followed by 4wks of recovery on a normal (4wkPO) or Ki20227-supplemented diet (4wkPO + Ki), and non-VE age-matched control mice. Each point represents one tumor nodule; 1-way ANOVA, ****P < 0.0001 (*n* = 3–7 mice). **(C)** Graph showing the fold-change in percentage of cells forming tumor spheres in the presence of TAMs, relative to without TAMs. Tumor cells from VE mice on normal (VE) or Ki20227 diet (VE+Ki) were stimulated with TAMs derived from non-VE old mice. Bars represent mean ± SE; 2-way ANOVA, ****P < 0.0001, N.S. not significant (*n* = 3–4 mice). **(D)** Graph showing the gene expression level of Sms (left panel) and APAO (right panel) in CD34^−^ tumor cells derived from VE mice on normal (VE) or Ki20227 diet (VE+Ki) and non-VE age-matched control mice. Each point represents one mouse; 1-way ANOVA, *P < 0.05 (*n* = 6–7 mice).

Previously we have shown that RETAAD tumors harbor a population of tumor-initiating cells (TICs) which can be detected by a sphere formation assay similar to the neurosphere assay. These TICs possess the unique capacity to form abundant tumor spheres *in vitro* in response to TAM stimulation [22]. This TAM-responsive TIC population normally emerges only in mice > 30 weeks of age, and is therefore a somewhat late event during the natural course of disease; however, treatment with the chemotherapeutic agent temozolamide drives early emergency of TAM-responsive TIC which may exacerbate tumor development. We hypothesized that surgical trauma also induced the emergence of the TAM-responsive TIC population which then contributed to tumor regrowth at the resected site and increased growth of metastases. To test this hypothesis we conducted VE on RETAAD mice at 10–15 weeks of age and characterized the behavior of their tumor cells four to six weeks later. This protocol is similar to the well-validated positive-margin resection model previously published [23]. The use of older mice also enabled us to recover sufficient cells from their relatively larger tumors to be able to assess their capacity to form tumor spheres *in vitro* as a measure of their TIC content. For the tumor sphere assays, eye tumors from recovered VE and non-VE animals were dissociated into single cells, and the CD34^−^ tumor cell population, containing the TAM-responsive TIC fraction [22], was enriched by flow cytometric removal of PDGFRα^+^ fibroblasts, CD31^+^ endothelial cells and CD45^+^ immune cells. CD11b^+^F4/80^+^ TAMs were isolated from within the CD45^+^ population from the tumors of old (>30 weeks) non-VE control mice. The CD34^−^ tumor cells were then cultured in suspension cultures to form tumor spheres with or without TAMs, at a ratio of 1:50 (TAM: tumor cell), reflecting the normal abundance of TAMs within the tumors [24] (Supplementary Figure S2A). As expected, cells derived from eye tumors of old non-VE mice (> 30 weeks) formed significantly more spheres after 5–7 days culture with TAMs than without TAMs, while tumor cells from young mice, age-matched to the VE group, did not (Figure 4A). Interestingly, cells derived from eye tumors of both the 4wkPO and 6wkPO VE group responded to the addition of TAMs and formed significantly more tumor spheres than in the absence of TAMs. The same results were seen when cells from metastases of VE mice were used: since the effect is similar, we pooled the data from the eye tumors and metastases into the same analysis to allow more robust statistical interrogation (Figure 4A). Thus surgical trauma promotes the emergence of a population of TAM-responsive TICs in both the recurrent tumor as well as the metastases.

We were also curious to know whether surgical trauma also affected the function of the TAMs in addition to increasing their number. Previously we showed that the stimulatory activity of TAMs depends on the production of TGFβ and polyamines via activation of Arginase 1 [22]. Thus we quantified the level of TGFβ and Arginase 1 transcripts in TAMs at 6wkPO, and found that surgical resection of the eye significantly increased the expression of both genes (Supplementary Figure S2B and S2C). TAMs from VE mice were significantly more stimulatory than TAMs from non-VE mice on tumor cells from old control mice (Supplementary Figure S2D), while when tumor cells from VE mice were used, the response was similar to TAMs from control and VE animals (Supplementary Figure S2E). This suggests that there is an increase in TAM activity after surgery, but only old mice harbor TICs that can respond, while young tumor cells are acquiring the ability to respond to TAMs and have yet to reach their full responsiveness.

The kinase inhibitor Ki20227 targets specifically the colony stimulatory factor-1 receptor (CSF-1R), which is important for macrophage survival and function [25]. Using this inhibitor we previously showed that inhibition of macrophage function prevented the induction of TAM-responsive TICs by temozolamide [22]. Here we asked whether inhibition of macrophages after surgical resection would similarly prevent the development of TAM-responsive TICs following VE. 10–15 week old mice that underwent VE were fed a normal or Ki20227-supplemented diet during their recovery for four to six weeks before cells from the primary tumor site and from metastases were harvested for sphere-forming assays. Ki20227 treatment of mice significantly reduced the density of macrophages in tumors compared to untreated VE mice at 4wkPO (Figure 4B). When tumor cells from Ki20227-treated animals were stimulated with TAMs from untreated non-VE old mice they no longer increase sphere formation in respond (Figure 4C), indicating that macrophage depletion by inhibition of CSF-1R prevents the development of the TAM-responsive TICs after surgery.

Previously we also showed that TAMs stimulate tumor sphere formation from TICs in RETAAD melanomas by producing polyamines via the Arginase 1 pathway: this observation would suggest that polyamine metabolism might be dysregulated in these tumors as has been reported for other tumor cell types [26]. To determine whether surgery similarly contributed to polyamine dysregulation and consequently droved tumor cells to exploit growth stimuli from TAMs, we quantified the expression of several enzymes in the polyamine metabolic/catabolic pathway within the CD34^−^ tumor cells. We found that transcripts for spermine synthase (Sms), which converts spermidine to spermine for final usage by the cell, were significantly more abundant in CD34^−^ tumor cells from VE animals compared to non-VE controls (Figure 4D left panel). The catabolic enzyme N-acetylpolyamine oxidase (APAO), which breaks down polyamines, was also increased post-surgery (Figure 4D right panel). This suggests that surgery increases the turnover of polyamine within the tumor cells, indicating that there is increased polyamine consumption and consistent with the increased tumor growth observed in these animals. When CD34^−^ tumor cells from Ki20227 treated animals were analyzed there was a significant reduction in Sms expression, but a further increase in APAO expression (Figure 4D). This indicates that depletion of the TAMs reduces the supply of polyamines leading to reduced expression of Sms while the increase in APAO expression further depletes polyamines within the tumor cells, both mechanisms leading to reduced tumor proliferation.

### Macrophage depletion reduces tumor growth after surgery

**Figure 5:**
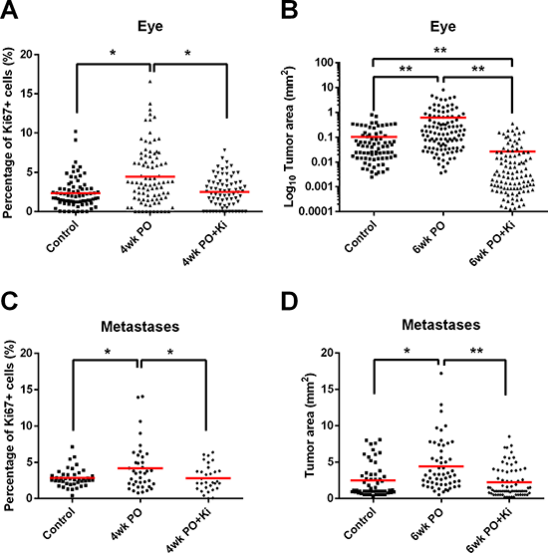
Macrophage depletion reduces tumor growth after surgery **(A)** Percentage of Ki67^+^ cells in relapsed eye tumors from VE mice on normal (VE) or Ki20227 diet (VE+Ki) compared with non-VE age-matched control mice. Each point represents one tumor nodule; 1-way ANOVA, *P < 0.05 (*n* = 4–6 mice). **(B)** Area of relapsed eye tumors from VE mice on normal (VE) or Ki20227 diet (VE+Ki) compared with non-VE age-matched control mice. Each point represents one tumor nodule; 1-way ANOVA, **P < 0.01 (*n* = 3–5 mice). **(C)** Percentage of Ki67^+^ cells in metastases from VE mice on normal (VE) or Ki20227 diet (VE+Ki) compared with non-VE age-matched control mice. Each point represents one tumor nodule; 1-way ANOVA, *P < 0.05 (*n* = 3–5 mice). **(D)** Area of metastases from VE mice on normal (VE) or Ki20227 diet (VE+Ki) compared with non-surgical age-matched control mice. Each point represents one tumor nodule; 1-way ANOVA, *P < 0.05, **P < 0.01 (*n* = 3–7 mice).

The above experiments showed that depletion of macrophages with Ki20227 inhibits the development of TAM-responsive TICs and reduces expression of the polyamine metabolic enzyme Sms, while increasing the expression of the polyamine catabolic enzyme APAO. We may therefore expect that macrophage depletion will affect tumor regrowth at the primary site, as well as the growth of metastases following surgery. Returning to the original experimental plan with VE performed on mice of one or three weeks of age, we now added Ki20227 treatment post-surgery. We found that Ki20227 treatment significantly reduced the percentage of Ki67^+^ proliferating tumor cells at 4wkPO relative to primary site tumors from untreated VE mice (Figure 5A), and that by 6wkPO this translated into significantly smaller tumor size (Figure 5B). The same effect was also observed for the metastases; significantly lower percentages of proliferating cells within tumors at 4wkPO (Figure 5C) and reduced tumor size at 6wkPO (Figure 5D). Interestingly, Ki20227 treatment not only abrogated the enhancement of tumor regrowth at the primary site that was associated with VE, but reduced the size of tumors to significantly below that of non-VE control animals (Figure 5B). In the case of the metastases, only the enhanced growth after surgery was abrogated by Ki20227 treatment (Figure 5D). This effect did not relate to differences in extent of macrophage depletion, as tumors at the primary site and metastases both showed approximately 75% reduction in macrophages density with Ki20227 treatment (data not shown). Thus our data show that macrophage depletion following surgical resection can significantly reduce the size of relapsed tumors at the primary site as well as limiting the enhanced growth of any metastases disseminated prior to surgery.

## DISCUSSION

In the RETAAD mice, disseminated tumor cells can be detected in various internal organs from 3 weeks of age [19]. However, our current data show that the development of metastases was unaffected even when the eyes were resected at 1 week of age: this suggests that tumor cells may disseminate much earlier than previously thought. While we did not formally exclude the possibility that the multiple lesions observed may have derived from independent transformation events, our previous SNP analysis strongly argues against this, instead indicating that the multiple lesions in RETAAD mice do originate from the primary eye tumor [19]. Thus dissemination is likely to have occurred during the hyperplastic lesion stage, supporting earlier observations in melanoma [27] and cervical cancer [28] patients. This has profound implications for cancer therapy. Since any therapeutic intervention will likely alter the microenvironments of disseminated tumor cells, as well as treating the cancer, it runs the risk of driving metastatic exit from a dormant state and initiating outgrowth. Indeed, surgery- associated metastatic escape from dormancy has been documented for several types of cancer [29].

In our current work we show that macrophages play an important role in tumor recurrence and metastatic outgrowth following surgical resection of the primary tumor. Macrophages are one of the first cell types to enter the postsurgical site and are essential for wound healing [20]. They are an important source of TGFβ which regulates critical steps of wound healing. TGFβ is also known to promote tumor growth. Accordingly, inhibition of TGFβ can prevent postoperative tumor relapse in a murine model of mesothelioma [30]. Thus it is surprising how little is known about the contribution of macrophages to the progression of cancer following surgery. We show here that the increased density of macrophages in both the relapsing tumor and the metastases of postoperative mice is associated with increased tumor proliferation and growth in the weeks following surgery. Macrophage depletion with Ki20227 reduced growth of tumors at the primary site significantly below even non-VE controls, indicating that resection combined with macrophage depletion might be an effective clinical approach to reduce postoperative local tumor recurrence. The anatomy of the orbital structure may have limited our ability to achieve complete removal of early locally-disseminated cells or increased shedding of primary tumor cells during surgery. Therefore individuals bearing tumors in sites more amenable to complete resection may see greater benefit if surgery is combined with macrophage depletion. The effect of macrophage depletion was also evident at the metastatic sites, supporting the use of systemic macrophage inhibition as opposed to a local approach at the wound site. A cautionary consideration in applying macrophage depletion relates to the role of macrophages in wound healing. Although we did not observe wound healing defects with Ki20227, preliminary experiments using clodronate liposome applied directly to the surgical site resulted in poor wound healing (data not shown). A possible explanation for this difference is that Ki20227 did not deplete the macrophages completely but rather limited the increase in macrophages following surgery, while clodronate liposome applied directly to the surgical site may have a more drastic effect on macrophage infiltration therefore preventing wound healing. Thus, further work will be necessary to identify optimal conditions for macrophage depletion before it can be used in the clinics.

Macrophages have previously been reported to regulate the metastatic process, promoting metastatic phenotypes in tumor cells [31], and regulating both intravasation of tumor cells into blood vessels [32] and their extravasation at the metastatic site [33]. However, these mechanisms did not seem to be of primary importance in the current study; where the total number of metastases was unaffected by either surgery or Ki20227 treatment (data not shown), presumably as a result of the early dissemination evident from the VE studies in 1 week old mice.

We propose that macrophages contribute to postsurgical tumor relapse and metastatic growth by stimulating a population of CD34^−^ TICs. The observation that tumor cells from young mice do not normally respond to macrophage stimulation, but can acquire the ability to respond after surgery, is similar to our previous observation in chemotherapy-treated young mice [22]. This supports the hypothesis that melanoma-initiating cells are flexible and can switch phenotypes [34] in response to a changing microenvironment. This observation substantially increases the complexity of therapeutic development. Previously we found that chemotherapy not only induced the tumor cells to change their responsiveness to macrophages, but that the macrophages from chemotherapy-treated mice also became more stimulatory, together resulting in a much greater induction of tumor sphere formation *in vitro*. In the current study, surgery also induced the appearance of the macrophage-responsive TICs and increased the stimulatory activity of the macrophages, reflected in increased TGFβ and Arginase 1 expression as well as their ability to stimulate control tumor cells to a greater extent than control macrophages (Supplementary Figure S2). However, tumor cells from the VE animals responded equally to macrophages from control and VE mice. This suggests that there may be multiple populations of tumor cells in older mice capable of responding to subtle changes in the macrophages, while younger tumor cells are acquiring new survival skills after surgery. Given the myriad of growth-stimulatory signals in the healing wound, responsiveness to macrophage stimulation is likely to contribute only partially to tumor recurrence, as can be seen by the inability of macrophage depletion to completely inhibit tumor regrowth. Similarly the presence of other immune cells such as neutrophils and myeloid-derived suppressor cells as well as endothelial cells and tumor-associate fibroblasts may also contribute to postsurgical tumor growth. Nevertheless, limiting the effect of macrophages will likely contribute to positive postsurgical outcomes.

Previously we have shown that the main stimulatory signals from TAMs are TGFβ and polyamines: here we reveal that surgery can also increase polyamine metabolism in the tumor cells which may drive their dependency on macrophages as a source of polyamines. Ki20227 treatment reduced expression of the metabolic enzyme, while increasing those for the catabolic enzyme, indicating that macrophage depletion may impact tumor growth by removing the source of polyamine. A number of polyamine inhibitors are currently in clinical trials and may add to the repertoire of adjuvant cancer therapies in the future [26, 35].

Surgery-related stress has also been associated with tumor relapse by directly enhancing tumor growth and modulating the immune microenvironment [10]. In our surgical setting, removal of the eyes may produce long-term stress that can modulate the activity of the macrophages and other immune cells and contribute to tumor growth. Interestingly, soluble factors known to be associated with surgical stress such as prostaglandins have also been shown to promote macrophage polarization to the M2 phenotype [36, 37]. Prostaglandins also produce immunosuppressive effects by reducing Th1 cytokines [38]. While ongoing efforts are focused on enhancing immune responses after surgery, for example by treatment with IL-12 [39, 40], or boosting NK cell functions [41, 42], understanding how immune subsets such as macrophages contribute to relapse could lead to the development of new therapies. Macrophage depletion, as a monotherapy, has already been demonstrated to be beneficial in both pre-clinical models [43] and in patients [44]. Our study highlights the feasibility of combining macrophage depletion with tumor resection to achieve better clinical outcomes.

## MATERIALS AND METHODS

### Mice

Animal care and experimental procedures were approved by the Singapore IACUC under protocol 120742. RETAAD mice were generated as described [45]. Male and female mice at 1, 3 and 10–15 weeks of age were subjected to visual enucleation and four to six weeks later were euthanized and necropsied. Male and female mice 30–35 weeks of age were used as aged controls. Metastases were identified visually during necropsy, removed and photographed with a ruler in the visual field. Tumor areas were measured using ImageJ. Eye tumors and selected metastases were fixed for immunohistochemistry.

### Surgical procedures (visual enucleation)

Mice were injected with Buprenorphine (0.2–1 mg/kg) and Enrofloxacin (20 mg/kg) prior to surgery and anaesthetized with isoflurane during the surgery. Both eyes were removed by gently cutting the connective tissues around the sclera and severing the optic nerve. The orbits were filled with sterile Gelfoam (Pharmacia and Upjohn) and the eye lids sutured together to close the wound. Following surgery, mice were treated with Buprenorphine and Enrofloxacin for three days. Mice operated on at 1 week of age were returned to the breeding cage after surgery before weaning at 3 weeks of age.

### Drug administration

Ki20227 (synthesized by GVKBio) was incorporated into the Harlan 2918 diet (Harlan) to give an estimated effective treatment dose of 30–40 mg/kg/day (drug/mouse weight). Mice were fed *ad libidum* for the duration of the experiment. Prior to weaning, pups were given Ki20227 dissolved in 0.05% methylcellulose by daily oral gavage at the same treatment dose as above. Upon weaning, mice were transferred to the Ki20227 diet.

### Immunohistochemistry

Formalin-fixed paraffin-embedded tumor sections (5 μm) were immunolabeled for S100B to identify melanoma cells, and for Ki67 to identify proliferating cells. Sections were dewaxed in xylene and heat-treated in Target Retrieval Solution (DAKO). Non-specific antibody binding was blocked by incubating sections with 3% (v/v) hydrogen peroxide, biotin and avidin block (DAKO) and 10% (v/v) normal goat serum (DAKO). Rabbit anti-S100B (DAKO; 1:4,000) and rat anti-Ki67 (DAKO; 1:40) antibodies were then applied and incubated overnight at 4°C. S100B labeling was revealed with anti-rabbit HRP (DAKO) and AEC peroxidase substrate (Vector Laboratories), while Ki67 labeling was revealed with biotinylated donkey anti-rat (Jackson Lab; 1:300) followed by alkaline phosphatase-conjugated streptavidin (Rockland Inc.; 1:2000) and Alkaline Phosphate Substrate Kit III (Vector Laboratories). To visualize immune cells within the tumor bed we use anti-S100B (DAKO), CD45-APC (Biolegend; 1:200) and F4/80-biotin (AbDserotec; 1:50) antibodies overnight at 4°C. Anti-rabbit-FITC (DAKO; 1:500) and streptavidin-PE (Invitrogen; 1:500) antibodies were used to reveal S100B and F4/80 labeling respectively.

### Cell selection

TIC and immune cell populations were harvested from the same mice for each experiment. Tumor cell sub-populations were sorted by flow cytometry using CD34-biotin (eBioscience), CD45-FITC, PDGFRα-APC and CD31-PECy7 (Biolegend) antibodies followed by streptavidin-PE. TAMs were isolated using CD45-FITC, CD11b-PECy7 (Biolegend), and F4/80-biotin, followed by streptavidin-PE.

### Cell culture

Tumors were dissociated with collagenase A (1 mg/ml) and DNase I (0.1 mg/ml; Roche) and cultured in stem cell medium [DMEM/F12 (1:1), 1% penicillin/streptomycin, B27 supplement (Invitrogen), 10ng/ml bFGF and 20ng/ml EGF (Peprotec)] at 4000 cells/cm^2^ in ultra-low attachment culture wells (Corning). Culture plates were left undisturbed during the culture period to avoid stimulating sphere formation by aggregation. Spheres greater than 30 μm in diameter were counted 5–7 days after cell seeding. TAMs were added at a ratio of 1:50 to tumor cells on the day of seeding and remained for the duration of the culture.

### Gene expression analysis

Cells were homogenized in TRIZOL (Qiagen). RNA was extracted using the Qiagen RNeasy Micro kit. cDNA was reverse transcribed (Roche Applied Biosystem reagents) and subjected to quantitative PCR with SYBR green (Bio-Rad) and specific primers. TGFβ (5′-GGCTACCATGCCAACTTCTG-3′ and 5′-G CTTGCGACCCACGTAGTAG-3′), Arginase1 (5′-CAA GACAGGGCTCCTTTCAG-3′ and 5′-GTAGTCAGTC CCTGGCTTATGG-3′), Sms (5′-CAACGTGCTGGT TCTGGATG-3′ and 5′-TTGATGAGGTCCAGGTGC AG-3′) and APAO (5′-ACTGCCAGTTCATCCAGGTG-3′ and 5′-GAGTCTCCATAAACTCCGACTCC-3′). Gene expression was normalized to GAPDH (5′-TGCGAC TTCAACAGCAACTC-3′ and 5′-ATGTAGGCCATGA GGTCCAC-3′).

### Statistical analysis

Graphs were generated and statistical analysis was carried out using Graphpad Prism 6 software. The tests applied are indicated in the figure legends: two-tailed *t* tests were used for comparisons between two groups; one-way ANOVA was used to compare multiple groups with one experimental parameter; and two-way ANOVA was used to compare multiple groups with two experimental parameters. When multiples tumor nodules were presented for each mouse, the statistical analyses were performed with the mean value from each mouse.

Additional experimental procedures can be found in the Supplemental section.

## SUPPLEMENTARY MATERIALS AND METHODS



## Abbreviations

APAO: N-Acetyl polyamine oxidase
CSF1R: Colony stimulatory factor-1 receptor
FGF: Fibroblast growth factor
HB-EGF: heparin-binding epidermal growth factor (EGF)-like growth factor
IL-12: Interleukin-12
NK cell: Natural killer cell
PDGF: Platelet-derived growth factor
PDGFRα: Platelet-derived growth factor receptor-α
Sms: Spermine synthase
TAMs: Tumor-associated macrophages
TGFβ: Transforming growth factor-β
TICs: Tumor-initiating cells
VE: Visual enucleation
