# Supplementary material for: Macrophage depletion reduces postsurgical tumor recurrence and metastatic growth in a spontaneous murine model of melanoma
Source: Oncotarget. 2015 Feb 13;6(26):22857–68. doi: 10.18632/oncotarget.3127 (PMC4673204; doi:10.18632/oncotarget.3127)
Supplement: Supplementary file 1 [file oncotarget-06-22857-s001.pdf]

SUPPLEMENTARY MATERIALS AND METHODS

Bone marrow-derived macrophages

Bone marrow cells were cultured in complete medium [DMEM, 10% FBS, 1% penicillin/streptomycin, 1% L-glutamine, 30% L929 cell-conditioned medium] for 5 days and polarized towards M1 (100ng/ml LPS; Sigma, and 20ng/ml IFN-γ; Miltenyi) or M2 (20ng/ml IL-4; Miltenyi) phenotypes with cytokines overnight.

SUPPLEMENTARY FIGURES

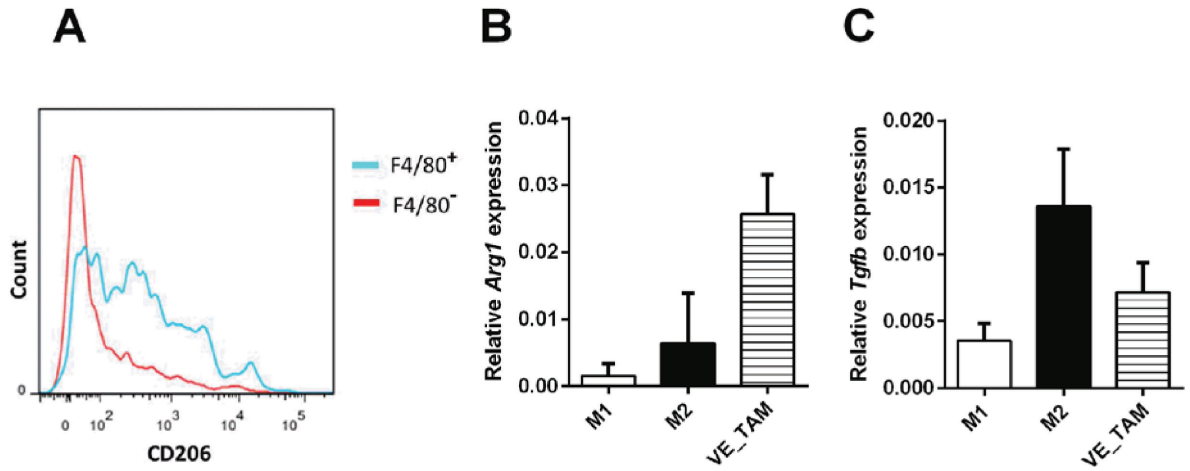

Supplementary Figure S1: Characteristics of macrophages after surgical resection. **(A)** Flow cytometry histogram showing F4/80+ TAMs from VE mice express the M2 marker CD206. **(B)** Graph comparing the gene expression level of Arginase 1 relative to GAPDH in bone marrow-derived macrophages polarized to the M1 and M2 phenotypes with TAMs derived from VE mice. Bars represent mean ± SD. **(C)** Graph comparing the gene expression level of TGFβ relative to GAPDH in bone marrow-derived macrophages polarized to the M1 and M2 phenotypes with TAMs derived from VE mice. Bars represent mean ± SD.

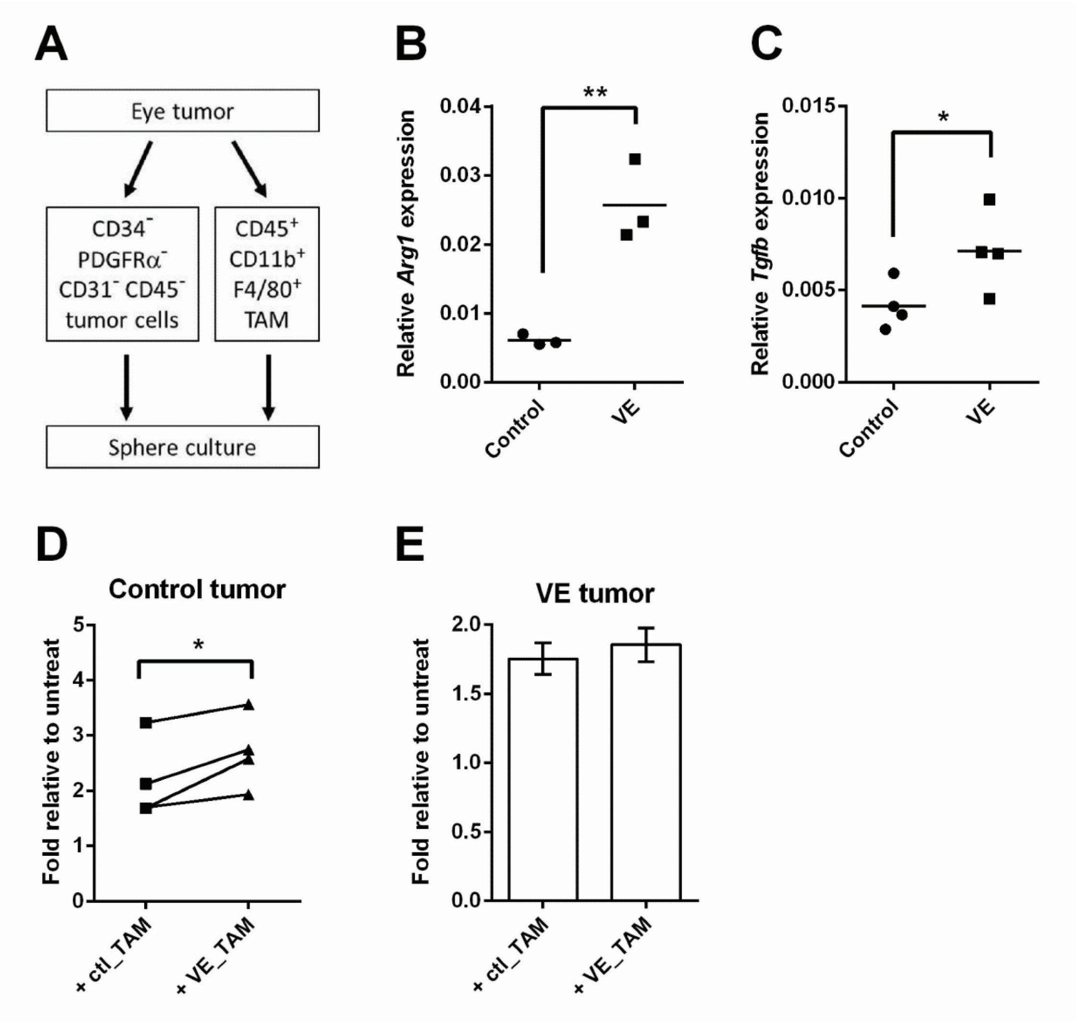

Supplementary Figure S2: **(A)** Schematic of tumor sphere culture procedure. **(B)** Graph comparing the gene expression level of Arginase 1 relative to GAPDH in TAMs derived from control and VE mice. Each point represent one mouse; unpaired two-tailed *t* test, $^{**}P < 0.01$ ($n = 3$ mice). **(C)** Graph comparing the gene expression level of TGFβ relative to GAPDH in TAMs derived from control and VE mice. Each point represent one mouse; unpaired two-tailed *t* test, $^{*}P < 0.05$ ($n = 4$ mice). **(D)** Graph showing the fold-change in percentage of tumor sphere formation when control tumor cells were cultured with TAMs derived from control (+ctl_TAM) or VE mice (+VE_TAM) relative to untreated. Each pair represent one set of experiment; paired two-tailed *t* test, $^{*}P < 0.05$ ($n = 4$ mice). **(E)** Graph showing the fold-change in percentage of tumor sphere formation when tumor cells from VE mice were cultured with TAMs derived from control (+ctl_TAM) or VE mice (+VE_TAM) relative to untreated. Bars represent mean ± SE.
